# Increase of Incidence and Mortality of Ovarian Cancer during 2003–2012 in Jiangsu Province, China

**DOI:** 10.3389/fpubh.2016.00146

**Published:** 2016-07-07

**Authors:** Zhimei Teng, Renqiang Han, Xingyu Huang, Jinyi Zhou, Jie Yang, Pengfei Luo, Ming Wu

**Affiliations:** ^1^Department of Epidemiology and Medical Statistics, School of Public Health, Southeast University, Nanjing, China; ^2^Department of Chronic Disease Control, Jiangsu Provincial Centre for Disease Control and Prevention, Nanjing, China

**Keywords:** ovarian cancer, incidence, mortality, trend analysis, China, Jiangsu

## Abstract

**Purpose:**

The objective of this study is to investigate and analyze the epidemiologic characteristics and time trends of ovarian cancer incidence and mortality in Jiangsu Province of China during 2003–2012.

**Method:**

Data were collected from eligible cancer registries in Jiangsu Province. Crude rates, age-specific rates, truncated age-standardized rate, and proportions of ovarian cancer were calculated. The Segi’s World Population was used to calculate age-standardized rates for world (ASW). Poisson distribution was used to analyze the differences between urban and rural areas. Joinpoint regression was performed to estimate the annual percent change (APC) of ovarian cancer incidence/mortality.

**Results:**

A total number of 4,401 new cases and 1,918 deaths were identified during period 2003–2012. The incidence and mortality ASW was 3.64/100,000 and 1.52/100,000, respectively. ASW of incidence was 4.48/100,000 in urban areas, while 3.04/100,000 in rural areas. The mortality of ASW was slight higher in urban areas than in rural areas. Age-specific incidence showed a peak at the age group of 60–64 years, whereas mortality peaked at age group of 65–69 years. A significant increase of incidence was observed from 2003 to 2006, with an APC of 34.0% (95% CI: 9.7, 63.7), the increasing rate declined since 2006 (APC = 3.3%, 95% CI: −3.5, 10.5). The mortality showed a gentle upward trend as compared with incidence, with an APC of 9.9% (95% CI: 7.7, 12.2) per year, continuously from 2003 to 2012. It is apparent that both incidence and mortality presented a rising trend in all areas, but urban were higher than that in rural areas.

**Conclusion:**

Ovarian cancer is a highly lethal disease which is becoming a significant public health problem in Chinese women. It is vital to improve the understanding of current status of ovarian cancer. Moreover, prevention and control policies should be formulated to reduce the disease burden of ovarian cancer in China.

## Introduction

Ovarian cancer is the sixth most common malignancy cancer in the world (1). It is one of the three major types of gynecological malignancy and ranks third after cervical cancer and uterine corpus cancer. Although the incidence and mortality of ovarian cancer are not very high as compared with common cancers, the absence of either specific symptoms or effective screening strategies caused more than 70% of patients were in advanced stage when diagnosed (2), which results in relatively high risk of recurrence and poor prognosis. According to report of Beard et al. (3), the relative 5-year survival rate for stage I ovarian cancer was 81%, however, for stage II, III, and IV was only about 59, 30, and 20%, respectively (3).

Ovarian cancer incidence has been rising continuously in the world, with 200,000 new cases diagnosed every year. International Agency for Research on Cancer (IARC) estimated that the world new cases and deaths of ovarian cancer were 238,700 and 151,900 occurred in 2012 worldwide, which accounted for 3.59 and 4.28% of all malignant cancer among women (4), and the number was higher than that in 2008, which was 225,000 cases and 140,000 deaths, respectively (5).

Similar as many common cancers, the risk of ovarian cancer varies across the world. Generally, higher incidence was observed in developed counties such as Northern Europe (11.3/100,000) and Northern America (10.7/100,000), while China (3.2/100,000) has been found with the lower incidence (6). But in recent years, the incidence of ovarian cancer in China showed an upward trend (7). However, few data have reported the situation of ovarian cancer in China.

Jiangsu Province is one of the developed areas and located in South-Eastern part of China. The population of Jiangsu is more than 80 million and 49.96% of which are women. With a large number population of women, gynecologic malignancy worth more public attention. Besides, Jiangsu is a high cancer risk area, according to the results of the 1990–1992 National Mortality Retrospective Sampling Survey, cancer mortality was 159.8/100,000 in Jiangsu and was about 50% higher than the national average (108.3/100,000) (8). Some papers have described the epidemiology of uterine corpus cancer and breast cancer, whereas no previous reported the incidence and mortality of ovarian cancer in Jiangsu Province yet.

The purpose of this study is to analyze and describe the epidemiologic characteristics and time trends of ovarian cancer in Jiangsu Province. It aims to improve the understanding of current status of ovarian cancer and to provide more scientific basis for the prevention of this disease in China.

## Materials and Methods

### Data Collection

The data of ovarian cancer during 2003–2012 were collected from the Jiangsu Provincial Cancer Registry. Jiangsu Provincial Cancer Registry is responsible for organizing, administrating, and supervising cancer registry works in different counties in the province. Cancer registry was first initiated in 1970s in several counties (9) and has been developed dramatically over the past years, now the registry system has covered the whole province. In our study, only 7 counties could provide integrated data since 2003, while eligible cancer registry areas gradually reached to 32 counties in 2012. Information of ovarian cancer patients, including personal information (name, age, birth date, phone number, address, etc.) and diagnostic information (onset date, diagnostic basis, etc.), were collected using standardized cancer registry card. From 2003 to 2012, the total population obtained in select registry areas was 176,104,111 person years (women accounted for almost 50%), which included urban and rural population of 73,955,792 and 102,148,319, respectively.

### Quality Control

Based on the protocol of assessment and recruitment criteria of data quality from the National Central Cancer Registry of China (NCCR), the MS-FoxPro, MS-Excel, and IARC-crg Tool were used for quality evaluation and data cleaning. Indices of reliability, validity, and integrity were checked by using the proportion of morphological verification (MV%), percentage of cancer cases identified with death certification only (DCO%), mortality to incidence ratio (M/I), and percentage of unknown basis of diagnosis (UB%) (10). In our research, the MV% of ovarian cancer was 77.54%, DCO% was 0.64%, UB% was 0.27%, and M/I was 0.44, which indicated that the quality of data was at reasonable level.

### Statistical Analyses

The 10th version of International Classification of Diseases (ICD-10) was used for case identification; the code for ovarian cancer is C56. We calculated the crude rate and age-specific rate (0–, 1–4, 5–84 sub-stratified by 5, and 85+ years) using the number of new cases/deaths divided by women population in registry areas during the same period. The Fifth Chinese National Population Census in 2000 and Segi’s World Population were used to calculate age-standardized rates for China (ASR) and world (ASW) (11). The truncated age-standardized rate (TASR) was calculated among 35–64 age group using world standard population. Poisson distribution was used to analyze the differences between urban and rural areas. The annual percent change (APC) was applied to describe the time trends of incidence and mortality from 2003 to 2012. Joinpoint regression software (Version 4.3.1.0) was used to identify significant changes and trends in ovarian cancer, which is available through the surveillance research program of the US National Cancer Institute (12). Analysis started with 0 and tested by fitting model with a maximum of 1 joinpoint. The slope of each line segment of the best-fitting model was expressed as the APC in the crude and ASW rate.

## Results

It could be observed in Table 1 that a total number of 4,401 new cases were identified in selected cancer registry areas within the period of 2003–2012, which accounted for 2.27% and ranked 11 of all malignancy cancers among female. The crude and ASW incidence was 5.03/100,000 and 3.64/100,000, respectively. For people aged 35–64 years, the TASR was 7.82/100,000. It could also be seen in Table 1 that the crude rate, ASW, and the TASR in urban areas were higher than in rural areas.

**Table 1:** **The incidence and mortality of ovarian cancer in Jiangsu, 2003–2012**.

Index		Women population	New cases/deaths	Crude rate (1/10^5^)	ASR (1/10^5^)	ASW (1/10^5^)	Cumulative rate (%)		TASR (1/10^5^) 35–64	Proportion (%)	Rank
							0–64	0–74			
Incidence	All	87,567,627	4,401	5.03	3.82	3.64	0.28	0.39	7.82	2.27	11
	Urban	50,779,425	2,249	6.11	4.70	4.48	0.34	0.47	9.34	2.61	10
	Rural	36,788,202	2,152	4.24	3.18	3.04	0.24	0.33	6.74	2.01	11
Mortality	All	87,567,627	1,918	2.19	1.54	1.52	0.11	0.18	3.17	1.56	14
	Urban	50,779,425	889	2.42	1.70	1.68	0.11	0.19	3.32	1.81	13
	Rural	36,788,202	1,029	2.03	1.43	1.42	0.11	0.17	3.06	1.40	14

The number of deaths (1,918) accounted for 1.56% of total female malignancies from 2003 to 2012. The crude mortality rate was 2.19/100,000, higher than ASW (1.52/100,000). Even though the mortality difference between urban and rural areas was less obvious than incidence, the various indicators showed that mortality in urban areas were slight higher than rural area.

**Figure 1:**
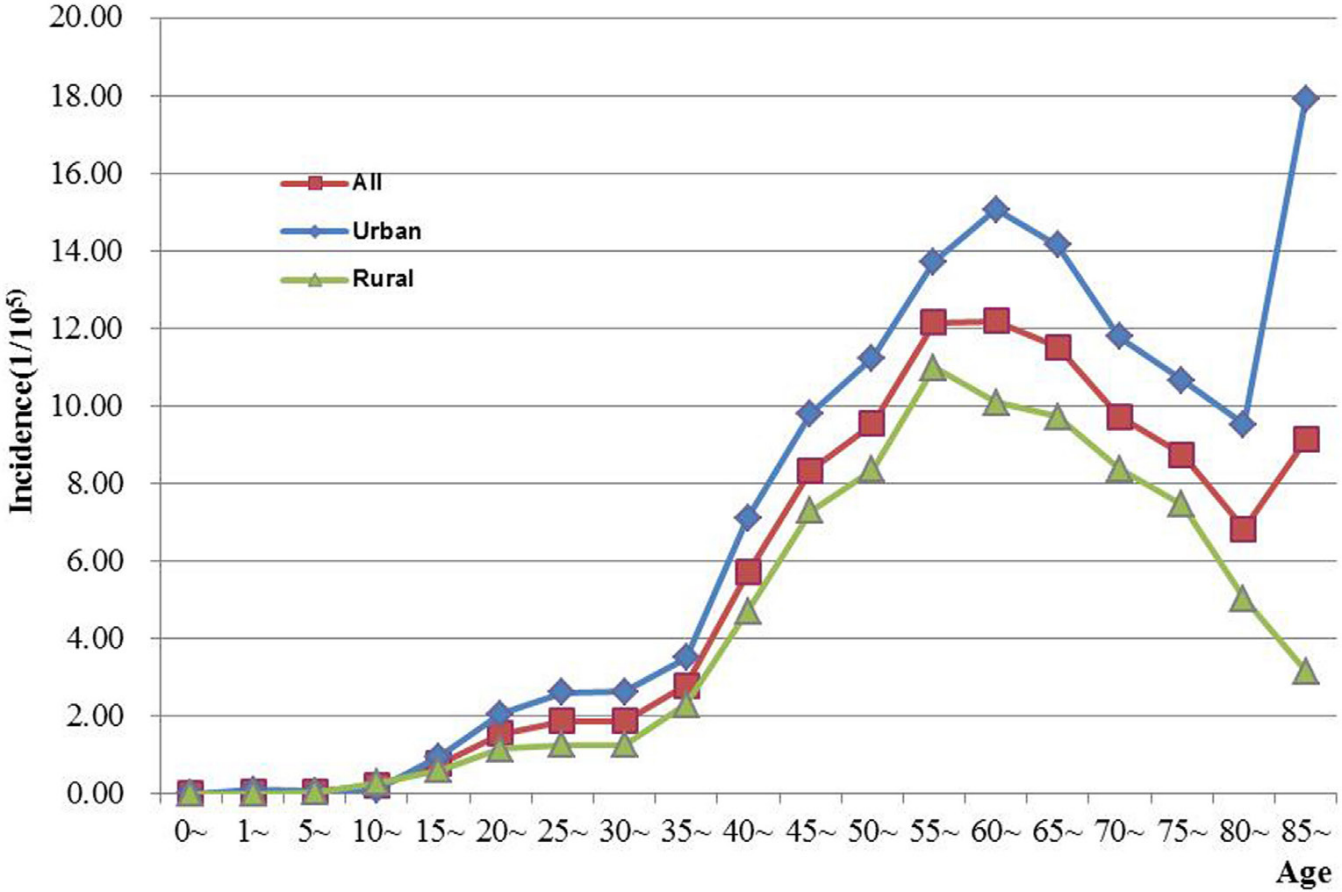
**Age-specific incidence of ovarian cancer in Jiangsu, 2003–2012**.

**Figure 2:**
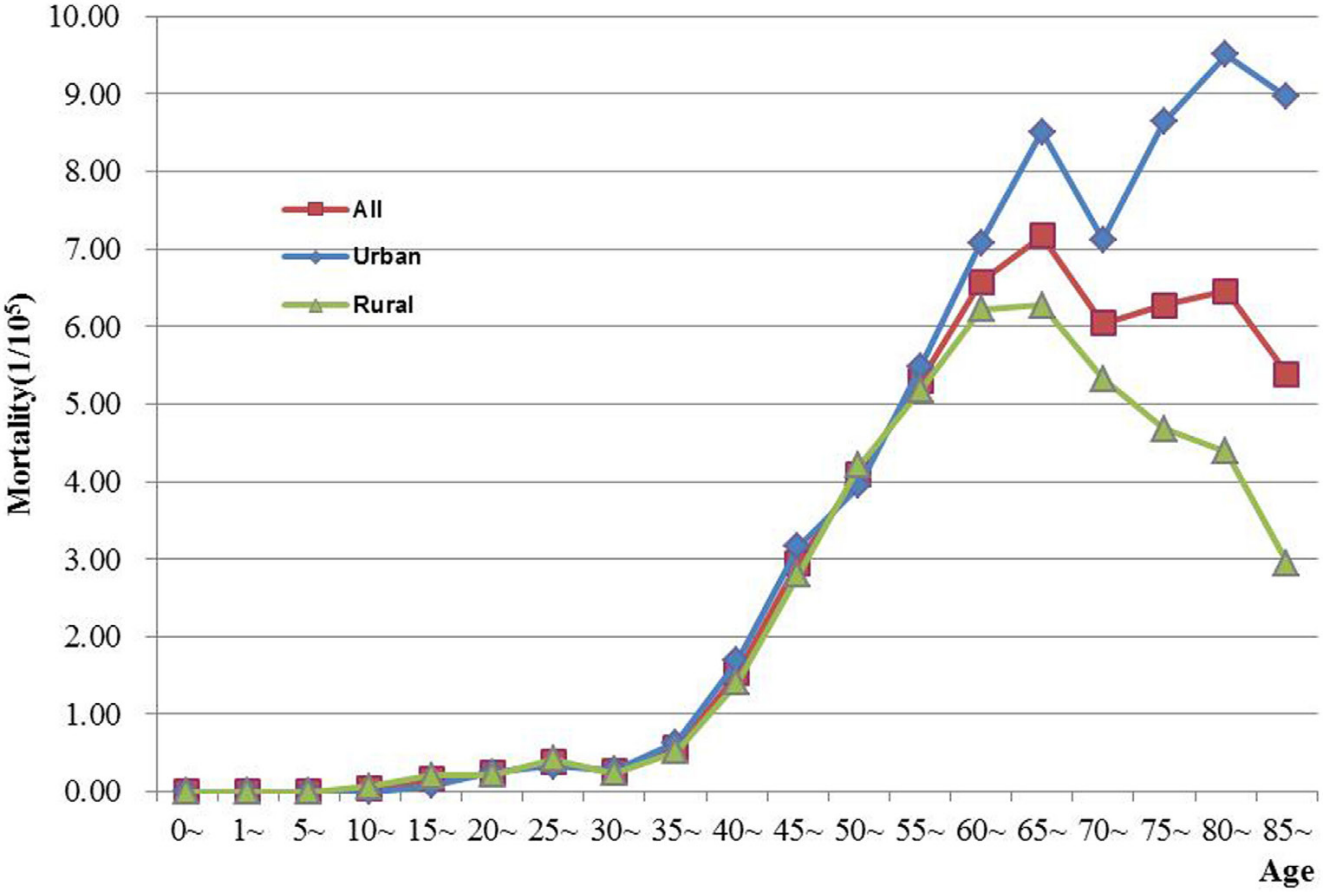
**Age-specific mortality of ovarian cancer in Jiangsu, 2003–2012**.

The age-specific incidence and mortality of ovarian cancer during the period of 2003–2012 in Jiangsu were presented in Table 2 (Figures 1 and 2). The ovarian cancer incidence was relatively lower before 40 years old, but increased rapidly after then and first peaked at the age group of 60–64 years (12.19/100,000). Afterward, the rate dramatically decreased to 6.82/100,000 at the age group of 80–84 years, but then increased again among women aged 85 years or older. The pattern was similar between urban and rural areas. But, the age-specific incidence was higher in urban area than in rural area. And, there was only one peak in rural area at the age group of 55–59 years. The trend of age-specific mortality was similar to its age-specific incidence, which also had two peaks. One peak was at the age group of 65–69 years and the second at age of 80–84. Similar patterns were found both in urban and rural areas. But only one peak was found in rural area. Comparing the rural area, the age-specific mortality in urban area was higher in most of age groups.

**Table 2:** **Age-specific incidence and mortality of ovarian cancer in Jiangsu, 2003–2012**.

Age group	Incidence						Mortality					
	All		Urban		Rural		All		Urban		Rural	
	Cases	Rate (1/10^5^)	Cases	Rate (1/10^5^)	Cases	Rate (1/10^5^)	Deaths	Rate (1/10^5^)	Deaths	Rate (1/10^5^)	Deaths	Rate (1/10^5^)
0~	0	0.00	0	0.00	0	0.00	0	0.00	0	0.00	0	0.00
1~	1	0.03	1	0.09	0	0.00	0	0.00	0	0.00	0	0.00
5~	2	0.05	1	0.06	1	0.04	0	0.00	0	0.00	0	0.00
10~	10	0.19	2	0.09	8	0.27	2	0.04	0	0.00	2	0.07
15~	43	0.76	23	0.95	20	0.61	9	0.16	2	0.08	7	0.21
20~	99	1.55	58	2.06	41	1.15	15	0.24	7	0.25	8	0.22
25~	123	1.86	78	2.60	45	1.25	25	0.38	10	0.33	15	0.42
30~	138	1.86	85	2.63	53	1.26	19	0.26	9	0.28	10	0.24
35~	213	2.79	111	3.49	102	2.29	43	0.56	20	0.63	23	0.52
40~	444	5.70	231	7.11	213	4.70	119	1.53	55	1.69	64	1.41
45~	595	8.32	288	9.79	307	7.29	211	2.95	93	3.16	118	2.80
50~	562	9.53	270	11.23	292	8.36	242	4.10	95	3.95	147	4.21
55~	648	12.14	308	13.72	340	10.99	283	5.30	123	5.48	160	5.17
60~	511	12.19	268	15.05	243	10.08	276	6.58	126	7.08	150	6.22
65~	390	11.50	193	14.15	197	9.72	243	7.17	116	8.51	127	6.27
70~	269	9.72	129	11.77	140	8.38	167	6.04	78	7.12	89	5.33
75~	184	8.74	90	10.66	94	7.45	132	6.27	73	8.64	59	4.68
80~	91	6.82	51	9.51	40	5.02	86	6.45	51	9.51	35	4.39
85~	78	9.13	62	17.92	16	3.15	46	5.39	31	8.96	15	2.95
2003–2012	4,401	5.03	2,249	6.11	2,152	4.24	1,918	2.19	889	2.42	1,029	2.03

**Figure 3:**
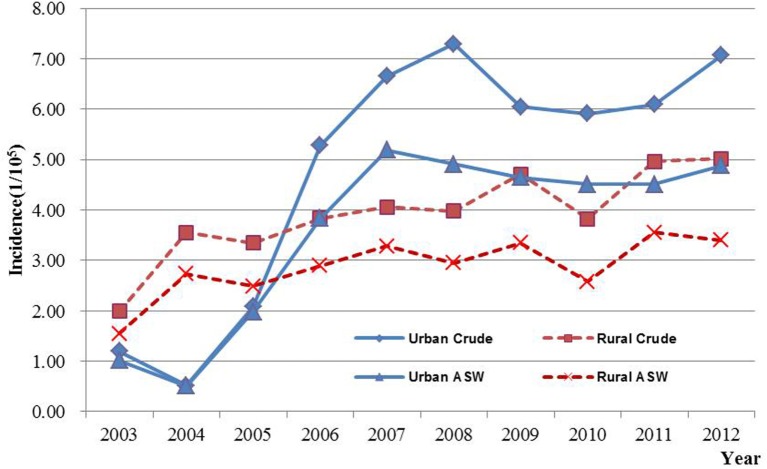
**The incidence time trends of ovarian cancer in Jiangsu, 2003–2012**.

**Figure 4:**
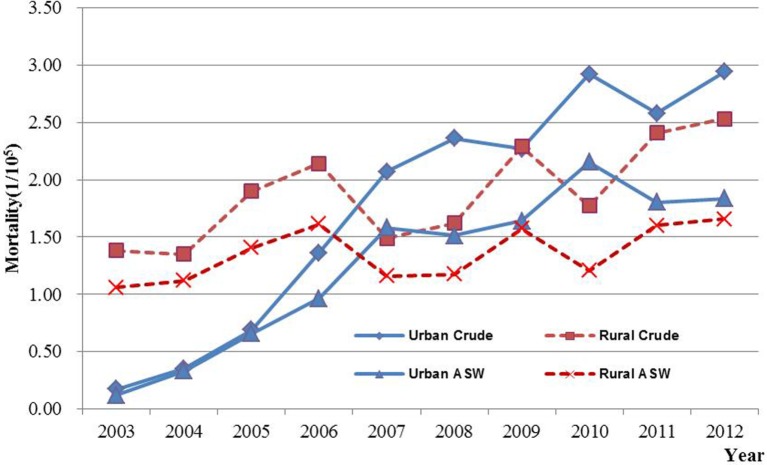
**The mortality time trends of ovarian cancer in Jiangsu, 2003–2012**.

Results of joinpoint regression analysis are presented in Table 3. Year 2006 was chosen as a jointpoint to separate the trend. A significant increase of incidence was observed from 2003 to 2006, with an APC of 34.0% (95% CI: 9.7, 63.7). However, the increasing rate declined since 2006 (APC = 3.3%, 95% CI: −3.5, 10.5). The mortality showed a gentle upward trend as compared with incidence, with an APC of 9.9% (95% CI: 7.7, 12.2). The temporal time trend for ASW incidence was similar to crude rate. But, the ASW for mortality was significantly increased by 7.2% (95% CI: 5.2, 9.2) per year, continuously from 2003 to 2012. The APC in urban areas was higher than that in rural areas for both incidence and mortality during the period of 2003–2012 (Figures 3 and 4). After age standardizing adjustment for Segi’s World Population, the APC of incidence and mortality became smaller in both urban and rural areas, but still with increasing trends. Moreover, the increasing trends of crude and ASW showed statistically significant except ASW mortality in rural areas.

**Table 3:** **The annual incidence and mortality and time trends for ovarian cancer in Jiangsu, 2003–2012**.

Index	Area	2003	2004	2005	2006	2007	2008	2009	2010	2011	2012	Trend 1		Trend 2		Total APC
												Period	APC% (95% CI)	Period	APC% (95% CI)	
**Incidence**																
Crude rate (1/10^5^)	All	1.83	3.05	3.14	4.63	4.89	5.56	5.16	4.81	5.54	5.90	03–06	34.0^a^ (9.7, 63.7)	06–12	3.3 (−3.5, 10.5)	11.1
	Urban	1.20	0.52	2.08	5.28	6.65	7.29	6.05	5.91	6.09	7.07	03–12	26.6^a^ (8.6, 47.6)			26.6
	Rural	1.99	3.55	3.35	3.83	4.06	3.98	4.71	3.82	4.96	5.02	03–12	7.5^a^ (3.2, 12)			7.5
	*P*	0.15	<0.01	0.06	0.01	<0.01	<0.01	0.02	<0.01	0.02	<0.01					
ASW (1/10^5^)	All	1.46	2.41	2.41	3.41	3.90	3.91	3.77	3.45	4.04	4.03	03–06	31.6^a^ (8.1, 60.3)	06–12	1.9 (−4.7, 8.9)	9.4
	Urban	1.02	0.51	1.98	3.84	5.19	4.91	4.64	4.51	4.51	4.88	03–07	67.4^a^ (4.4, 168.4)	07–12	−2.2 (−29.9, 36.6)	22.9
	Rural	1.55	2.73	2.48	2.89	3.29	2.95	3.34	2.58	3.55	3.40	03–12	5.9^a^ (1.2, 10.8)			5.9
	*P*	0.23	<0.01	0.42	0.01	<0.01	<0.01	<0.01	<0.01	<0.01	<0.01					

**Mortality**																
Crude rate (1/10^5^)	All	1.14	1.19	1.70	1.71	1.68	1.97	2.28	2.32	2.50	2.71	03–12	9.9^a^ (7.7, 12.2)			9.9
	Urban	0.17	0.35	0.69	1.36	2.07	2.36	2.27	2.92	2.58	2.94	03–07	87.6^a^ (73, 103.4)	07–12	4.8 (−1.0, 11)	34.2
	Rural	1.38	1.35	1.90	2.14	1.49	1.62	2.29	1.77	2.41	2.53	03–12	5.9^a^ (1.6, 10.4)			5.9
	*P*	<0.01	<0.01	<0.01	0.02	0.13	0.01	0.95	<0.01	0.49	0.10					
ASW (1/10^5^)	All	0.90	0.99	1.31	1.25	1.29	1.35	1.59	1.62	1.70	1.73	03–12	7.2^a^ (5.2, 9.2)			7.2
	Urban	0.12	0.33	0.66	0.97	1.58	1.51	1.64	2.15	1.80	1.83	03–06	111.5^a^ (72, 160)	06–12	8.2^a^ (0.9, 16.0)	30.4
	Rural	1.06	1.12	1.41	1.61	1.16	1.18	1.57	1.21	1.60	1.66	03–12	3.5 (−0.3, 7.5)			3.5
	*P*	<0.01	<0.01	0.05	<0.01	0.14	0.10	0.78	<0.01	0.23	0.27					

*^a^The annual percent change (APC) is statistically significantly at alpha = 0.05; p < 0.05 means statistically significant*.

## Discussion

Ovarian cancer is one of the most lethal gynecological malignancies. The exact etiology of ovarian cancer is still unclear, but it has been correlated with obesity, diet, lifestyle, environment, socioeconomic status, reproductive history, oral contraceptive, family history, and genetic factors (13).

Similar to many other types of cancer, there is significant geographic disparity in ovarian cancer incidence and mortality (14). According to the GLOBOCAN 2012 database, the incidence and mortality in developed areas was 9.1/100,000 and 5.0/100,000, respectively, which was higher than those in less developed areas (5.0/100,000 and 3.1/100,000) (4). Higher incidence areas were observed in Europe, North America, and lower in Asia, Brazil, Mexico, with approximately four times between the highest and the lowest incidence regions; besides, the mortality was over three times between the corresponding areas (14–16).

Similar variations have been described between different races by other studies (17). Morris et al. (18) reported that ovarian cancer incidence was higher in White (12.8/100,000) than Black (9.8/100,000) women (18). While compared with White, the Africa-American women were more likely to have higher mortality, due to the lack of sufficient diagnostics and sophisticated treatments, which usually led to presents more advanced cancers and shorter disease-free survival (19–22). The disparity of ovarian cancer in different world regions and races suggested that genetic factors and environmental factors, such as socioeconomic conditions, lifestyle, and other risk factors, may ultimately determine the risks (2).

China is one of the developing countries with the lowest incidence and mortality among selected areas (23), but an upward trend was observed from 2003 to 2007 (24). In this study, APC in 2006–2012 (APC = 3.3%, 95% CI: −3.5, 10.5) had a big change as compared with 2003–2006 (APC = 34.0%, 95% CI: 9.7, 63.7), but a significant rise in the pace was also observed in general with an APC of 11.1%. Our result was similar to a previous study, which observed a significant rising trend of ovarian cancer incidence during 1999–2006, while a drop happened during 2006–2010 for urban women, and a continuous rise was observed in rural women (6). In recent years, in spite of the combination of docetaxel, intraperitoneal cisplatin hyperthermic perfusion chemotherapy and hyperthermia to treat advanced ovarian cancer and could reduce the number of deaths (25); lack of early diagnosis is a main factor that leads to increasing mortality rates, which also makes ovarian cancer remaining the most important cause of gynecological cancer. During the past years, people living standard rise ceaselessly with the rapid economic growth and lifestyle have been greatly changed. The consumption of meat and fat has been tremendously increased, which is directly associated with risk of obesity, higher BMI and WC (26). Compared with 2002, rates of overweight and obesity raised 7.3 and 4.8% among Chinese residents aged more than 18 years in 2012 (27). A meta-analysis estimated that compared with women with a body mass index in the “healthy” range (BMI 18.5–24.9), the risk of epithelial ovarian cancer among obese (BMI of 30 or more) and overweight (BMI 25–29.9) women had 30 and 16% increased risk, respectively (28). With the social and economic development, mental activity is gradually replacing physical activity lifestyle which becomes the main way of our daily life. In a meta-analysis study, compared with women who described to the least exercise in daily life, the overall risk of ovarian cancer was 20% declined among women with recreational physical in both case–control and cohort studies (29).

The risk differences between urban and rural areas were also found in other counties. In Egypt, it was reported around two times higher incidence of ovarian cancer in urban areas compared with rural areas (30). A report in China showed the incidence of ovarian cancer in urban areas was 9.37/100,000 while in rural areas was 5.02/100,000, which was similar to our results (31). Given the fact that women in a specific area are genetically similar, therefore most of the risk differences could be explained by socioeconomic status, environment, and lifestyles.

The age-specific incidence was observed to rise with age, and reached its first peak at age of 60–64 years, and second peak among 85− years old. The same tendency of ovarian cancer shared in some Chinese reports (24, 31). Influence of the hormonal factors on the development of ovarian cancer might differ by different age group. In terms of etiology, ovarian cancer is not actually a hormonally related cancer; it is not under direct stimulatory effects of estrogen (30). Ovarian cancer development is more related to risk factor that causes chronic inflammation, related to “incessant ovulation” (32, 33). However, hormones may affect the development of ovarian cancer to some degree. A strong inverse relationship was observed between each live birth and ovarian cancer. Compared with nulliparae, the OR for ovarian cancer was 0.43, 0.30, and 0.18 for women whose parity was 1–2, 3–5, and 6–, respectively (34). This study also found menopausal status was associated with increased risk of disease (OR = 2.15, 95% CI 1.21, 3.83) (34). In addition, the combined oral contraceptive (OCs) conferred a protective crucial feature of epithelial ovarian cancer and was the most significant from a public health perspective (35). The lifelong number of menstrual cycle has been found to link with ovarian cancer, which suggested that ovulation is implicated and may subsequent cause ovarian carcinogenesis, but parity and OCs seems to play more important roles than that of other factors (36).

The limitations of our study should be acknowledged. First, the selected registries might not adequately represent the whole province especially in the beginning. Second, detail information of cancer cases, such as cancer stage and histological type, was not obtained in most registries; hence, we were not able to further analyze crude rates among stage and histological type, etc. Third, not all cancer registries are the members of International Association of Cancer Registration (IACR), and data were not included in Cancer Incidence in Five Continents yet.

In summary, ovarian cancer incidence has been significantly increased in Jiangsu in recent years, which also reflect the situation in China. Although the incidence and mortality are relatively low, considering the huge population, health education, and promotion of ovarian cancer have become increasingly important with respect to the prevention of ovarian cancer in China.

## Author Contributions

ZT and RH wrote original article and data analysis. MW revised the paper. The other authors monitored data and reviewed the paper.

## Conflict of Interest Statement

The authors declare that the research was conducted in the absence of any commercial or financial relationships that could be construed as a potential conflict of interest.
